# Épidémiologie des infections à VIH, hépatites B et C et interventions prioritaires pour l’élimination de l’hépatite B: investigation dans l’Extrême-Nord du Cameroun

**DOI:** 10.11604/pamj.2026.53.45.46297

**Published:** 2026-01-29

**Authors:** Joseph Fokam, Aristide Dama, Basile Yaba, Toussaint Malama, Paul Tjek, Joelle Nounouce Bouba Pamen, Ousmane Diaby, Rogers Ajeh Awoh, Malachie Manaouda

**Affiliations:** 1 Central Technical Group, National Aids Control Committee, Ministry of Public Health, Yaoundé, Cameroon,; 2 University of Buea, Faculty of Health Sciences and Chantal Biya International Reference Centre for Research on HIV/AIDS Prevention and Management, Yaoundé, Cameroon,; 3 Regional Technical Group, National Aids Control Committee, Far-North Regional Delegation of Public Health, Maroua, Cameroon,; 4 Department of Pharmacy, Drug and Laboratories, Ministry of Public Health, Yaoundé, Cameroon,; 5 Global Funds and Partners Grants Coordination Unit, Ministry of Public Health, Yaoundé, Cameroon,; 6 Department of Family Health, Ministry of Public Health, Yaoundé, Cameroon,; 7 Central Administration, Ministry of Public Health, Yaoundé, Cameroon,; 8 Faculty of Medicine and Biomedical Sciences, University of University I, Yaoundé, Cameroon,; 9 Faculty of Economics and Management Sciences, University of Yaoundé II, Soa, Cameroon

**Keywords:** VIH/SIDA, hépatite virale B, hépatite virale C, triple élimination, Cameroun, HIV/AIDS, hepatitis B virus, hepatitis C virus, triple elimination, Cameroon

## Abstract

**Introduction:**

dans le cadre de la triple élimination du VIH, des virus de l'hépatite B et C, une meilleure compréhension du fardeau local de ces infections est essentielle pour orienter les interventions ciblées. Cette étude vise à déterminer le profil épidémiologique du VIH, des hépatites virales B (VHB) et C (VHC) dans le département du Mayo-Tsanaga à l'Extrême-Nord du Cameroun.

**Méthodes:**

étude transversale de surveillance épidémiologique réalisée du 1^er^ août au 20 septembre 2024. Un échantillonnage consécutif a été effectué. Après obtention du consentement éclairé, chaque participant a rempli un questionnaire standardisé et a été testé pour le VIH, le VHB et le VHC selon l'algorithme national. Les données ont été analysées avec Excel 2019 et Power BI, avec un seuil de significativité p<0,05.

**Résultats:**

au total, 3188 participants (60% de femmes, âge médian 34 ans [IQR 20-46]) ont été inclus. Parmi eux, 396 (12,4%) ont été testés positifs à au moins une infection: VIH 0,41% (n = 13), VHB 11,7% (n = 373), VHC 0,31% (n = 10). Pour le VIH, une majorité de femmes (77%), concentrée dans la tranche de 25-34 ans. Pour le VHB, on avait une positivité élevée au-delà de 55 ans (27%), femmes 63,6%, pas de différence significative selon le sexe (p = 0,138). Pour le VHC, on avait une distribution homogène, sans différence significative selon âge ou sexe.

**Conclusion:**

nos résultats suggèrent une possible transition vers l'élimination du VIH et du VHC dans cette population au vu de leur faible séropositivité. En revanche, la forte positivité du VHB appelle à renforcer la stratégie vaccinale couplée au dépistage et à la sensibilisation ciblant prioritairement les jeunes.

## Abstract (en)

**Introduction:**

with the aim of guiding the strategic response to major viral infections, this study aimed to determine the epidemiological profile of HIV, hepatitis B virus (HBV), and hepatitis C virus (HCV) in the Far North region of Cameroon.

**Methods:**

we conducted a cross-sectional epidemiological surveillance study in the Mayo-Tsanaga Division and surrounding localities in the Far North region of Cameroon from August 1 to September 20, 2024. After obtaining informed consent, each participant was given a standard questionnaire and tested for the three infections (HIV, HBV, and HCV) in accordance with the national testing algorithm. The collected data were analyzed using Excel 2019 and Power BI software, with a statistical significance threshold set at p < 0.05.

**Results:**

a total of 3,188 participants were tested (60% female, median age 34 years [IQR 20-46]), of whom 396 tested positive overall, including 13 who were HIV-positive, 373 who were HBV-positive, and 10 who were HCV-positive. According to each type of infection, HIV, HBV and HCV seropositivity rates were 0.41% (13/3,170), 11.7% (373/3,188) and 0.31% (10/3,188), respectively. Despite the high endemicity of HBV (>8%), no statistically significant differences were observed by age or sex (p > 0.05).

**Conclusion:**

the low prevalence rates (<1%) of HIV and HCV suggest that their elimination is effectively underway among populations in the Far North region of Cameroon. However, the high endemicity of HBV indicates ongoing transmission and underscores the urgent need to prioritize vaccination as the primary preventive intervention toward elimination.

## Introduction

Selon l'Organisation mondiale de la Santé (OMS), l'infection à VIH demeure une urgence de santé publique d'envergure mondiale, avec des défis majeurs principalement dans les pays à ressources limitées [1]. En mi-2024, environ 40 millions de personnes vivaient avec le VIH au niveau mondial dont près des deux tiers en Afrique subsaharienne. D'après les données de 187 pays publiées par l'OMS, environ deux milliards (près du tiers de la population mondiale) ont déjà contracté le virus de l'hépatite B (VHB), avec au total 350 millions de personnes vivant une infection chronique du VHB. Un nombre de décès croissant de 1,1 million en 2019 à 1,3 million en 2022 parmi lesquels 83% sont attribuables au VHB et 17% au virus de l'hépatite C (VHC). D'après l'Agence des Nations Unies pour la lutte contre le VIH/SIDA (ONUSIDA), la tranche d'âge de 15 à 49 ans est la plus concernée avec un pic observé entre 15 et 24 ans, et particulièrement élevé chez les jeunes filles (2,3% de séroprévalence en moyenne), avec des taux encore préoccupants dans les pays en voie de développement [2].

Le Cameroun, comme la plupart des pays de l'Afrique subsaharienne, a une population essentiellement jeune (âge moyen autour de 20 ans); l'explosion démographique de la jeunesse le place ainsi en situation de vulnérabilité face aux infections virales. Avec une prévalence nationale du VIH située entre 2,7% (selon EDS 2018) et 3,1% (CAMPHIA 2017/2018), de 8% pour le VHB et moins d'1% pour le VHC en populations générales. Il convient de surveiller l'évolution de ces infections au niveau communautaire pour des actions probantes [2]. Une telle surveillance est utile en ce qu'elle permet d'orienter les politiques de prévention et de mettre en œuvre des interventions ciblées [3-5]. De plus, suivant l'approche de triple élimination de ces infections à travers l'intégration des services offerts aux populations, il convient de définir une échelle de priorité afin d'optimiser les interventions selon les ressources disponibles pour les programmes de santé publique répondant aux défis et enjeux en Afrique subsaharienne [2].

Dans l'optique de renforcer la stratégie nationale de lutte contre ces infections virales, il convient d'actualiser les données épidémiologiques dans ces localités du pays sur le VIH, le VHB et le VHC et d'informer les décideurs sur des actions prioritaires afin de contribuer aux efforts pour l'atteinte de l'élimination. L'objectif de cette étude était de déterminer la séropositivité des infections à VIH, VHB et VHC au sein des communautés vivant dans le département du Mayo-Tsanaga, région de l'Extrême-Nord du Cameroun.

## Méthodes

### Conception de l'étude

Une étude transversale de surveillance épidémiologique a été réalisée du 1^er^ août au 20 septembre 2024 au sein des communautés du département de Mayo-Tsanaga dans la région de l'Extrême-Nord du Cameroun.

### Site de l'étude

L'étude s'est étendue à la collecte des données dans sept formations sanitaires du département du Mayo Tsanaga sur la base de l'accessibilité géographique et de leur fréquentation et elles représentent toutes les échelles de la pyramide sanitaire disponibles au sein du département choisi. Il s'agit de: Hôpital Régional Annexe de Mokolo, Hôpital de District de Mokolo 2, Centre Médical d'Arrondissement de Mokong, Centre de Santé Intégré d'Ouro Tada, Centre Médical d'Arrondissement de Minawao, Centre de Santé Intégré de Tchouvouk, Centre de Santé Intégré de Ziling.

### Enrôlement des participants

Avant la mise en œuvre de l'étude, les autorisations administratives ont été obtenues à partir du Ministère de la Santé Publique du Cameroun (D/MINSANTE/07/2024). Le consentement éclairé de chaque participant a été obtenu après administration de la fiche d'information qui était expliquée en langue locale (Mafa et Fulani essentiellement). Cette fiche expliquait les objectifs de l'étude, les modalités de participation et la procédure. Ceci était fait par des personnels formés à cette fin. Nous avons procédé à un échantillonnage consécutif de tout patient éligible vivant dans le département du Mayo-Tsanaga ou dans les localités riveraines, ayant été reçu dans les hôpitaux sélectionnés au cours de la période d'étude et qui avait donné son consentement éclairé pour la participation à l'étude. Chaque patient recevait un code d'identification unique d'anonymat et les données recueillies ont été conservées de façon confidentielle.

### Algorithme de dépistage des infections à VIH, HVB et HVC

Le test du VIH a été réalisé selon l'algorithme en série sur la base des tests préqualifiés de l'Organisation mondiale de la Santé basés sur des principes d'immuno-chromatographie, avec comme premier test hautement sensible pour le screening de tout cas réactif (*Determine HIV1/2*) et comme second test hautement spécifique (*Shanghai HIV antibody rapid test*) pour la confirmation des cas réactifs en test 1. Tout échantillon non-réactif au test 1 était défini comme séronégatif; tout échantillon réactif consécutivement aux tests était ainsi défini comme séropositif.

Les tests des hépatites virales B et C ont été réalisés sur la base des tests préqualifiés de l'Organisation mondiale de la Santé basés sur des principes d'immuno-chromatographie, avec des sensibilités et spécificités standards. Le biomarqueur ciblé pour le screening du VHB était l'antigène de surface du virus (AgHBs), et le marqueur cible pour le VHC était l'anticorps dirigé contre le virus (anticorps Anti-HCV). Tout échantillon réactif à l'AgHBs était défini comme séropositif, traduisant ainsi la présence de l'infection au VHB. Tout échantillon réactif à l'Anti-HCV était défini comme séropositif, traduisant ainsi la présence d'anticorps au VHC. VIH: test rapide 1 (Determine HIV1/2) pour dépistage; test rapide 2 (Shanghai HIV antibody rapid test) pour confirmation; VHB: détection rapide de l'antigène de surface du VHB (AgHBs); VHC: détection rapide d'anticorps du VHC (Anti-VHC). La séropositivité est définie par la réactivité aux tests rapides correspondants à chacune des infections y afférentes.

### Analyses statistiques et interprétation des données

Les données ont été saisies à partir de questionnaires standardisés et, analysées à l'aide de Microsoft Excel 2019, SPSS et Power BI. Une description statistique initiale a été réalisée (effectif, proportion); la comparaison des proportions selon l'âge et le sexe a été faite pour identifier les facteurs indépendamment associés à la séropositivité au VIH, VHB et VHC par régressions logistiques binaires, en incluant les variables « sexe » et « tranche d'âge ». Les Odds Ratio (OR), intervalles de confiance à 95% (IC95%) et p-values ont été rapportés. Le seuil de significativité était fixé à p < 0,05.

### Considérations éthiques

Cette étude a été réalisée sous l’autorisation du Ministère de la Santé Publique du Cameroun et dans le cadre de la surveillance épidémiologique de la grande campagne de santé de Mayo-Tsanaga en 2024. Avant l’enrôlement, un consentement éclairé a été obtenu de tous les participants; le prélèvement sanguin a été réalisé par un personnel qualifié; les résultats ont été traités avec confidentialité et anonymat, puis rendus gratuitement à tous les participants.

## Résultats

### Description de la population

L'analyse a porté sur un échantillon de 3188 individus testés pour le VIH, le VHB et le VHC. La répartition était de 60% de femmes (n = 1921) et 40% d'hommes (n = 1267), avec un âge médian de 34 ans [IQR: 20-46] (Tableau 1).

**Tableau 1:** répartition des séropositifs par infection et sexe

Infection	Cas positifs	Prévalence (%)	Femmes	Hommes
VIH	13	0,41	10 (77%)	3 (23%)
VHB	373	11,7	237 (64%)	136 (36%)
VHC	10	0,31	5 (50%)	5 (50%)

**Figure 1:**
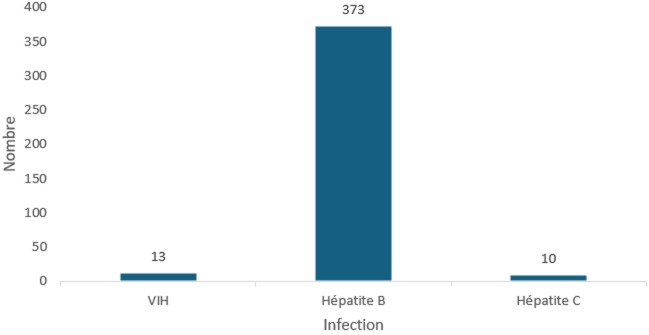
nombre de personnes testées positives par infection

Au total, 13 personnes ont été testées positives au VIH, soit un taux de séropositivité au dépistage de 0,41% (13/3188) parmi lesquelles 23% d'hommes contre 77% de femmes. Le taux de séropositivité au VHB était de 11,7% (373/3188) dont 36% chez les hommes et 64% chez les femmes. Le taux de séropositivité au VHC était de 0,31% (10/3188). Ce taux de séropositivité au VHC indique une faible endémicité (i.e. inférieure à 2%) au sein de la population d'étude. Par ailleurs, tous les cas positifs au VIH, VHB ou VHC ont été systématiquement liés aux services de prise en charge, soit un lien au traitement de 100% (Figure 1).

### Répartition de la séropositivité au VIH par tranche d'âge et par sexe (**Tableau 2**)

**Tableau 2:** répartition des personnes testées positives au VIH par tranche âge et sexe

Tranche d'âge (années)	Sexe	
	Hommes	Femmes
**15-24**	0 (0%)	2 (20%)
**25-34**	1 (33,33%)	4 (40%)
**35-44**	1 (33,33%)	2 (20%)
**45-54**	0 (0%)	1 (10%)
**≥55**	1 (33,33%)	1 (10%)
**Total**	**3** (100%)	**10** (100%)

La répartition des personnes testées positives au VIH par tranche d'âge révèle que la tranche d'âge la plus représentée était celle des 25-34 ans (38,5 % des cas), suivie des 35-44 ans (23,1%).

### Répartition de la séropositivité au VHB par tranche d'âge et par sexe

Le Tableau 3 ressort la répartition par tranche d'âge et par sexe des patients VHB positifs. Nous constatons que les femmes sont les plus représentées avec le taux de 64% (n = 237) comparé à 36% (136) d'hommes. La distribution semble homogène dans les différentes tranches d'âge avec un pic dans la tranche = 55 ans (27%).

**Tableau 3:** répartition des personnes testées positives à l'hépatite virale B par tranche âge et sexe

Tranche d'âge (années)	Sexe	
	Hommes	Femmes
15-24	23 (17%)	37 (16%)
25-34	34 (25%)	52 (22%)
35-44	27 (20%)	61 (26%)
45-54	15 (11%)	22 (10%)
≥55	37 (27,2%)	65 (27%)
**Total**	136 (100%)	(100%)

### Répartition de la séropositivité à l'hépatite virale C par tranche d'âge et par sexe

La séropositivité au VHC était de 0,31% (n=10) dont 50% de femmes et 50% d’hommes, justifiant ainsi une distribution similaire de la faible circulation du VHC au sein des cibles populationnelles selon le sexe (Tableau 4).

**Tableau 4:** nombre de personnes testées positives à l'hépatite virale C par tranche âge et sexe

Tranche d'âge (années)	Sexe	
	Hommes	Femmes
**15-24**	0 (0%)	0 (0%)
**25-34**	1 (20%)	1 (20%)
**35-44**	1 (20%)	3 (60%)
**45-54**	1 (20%)	1 (20%)
**≥55**	2 (40%)	0 (0%)
**Total**	**5** (100%)	**5** (100%)

Prenant en compte les trois infections suivant l'approche de triple élimination et de priorisation en matière d'intégration de services, aucune variation statistiquement significative de séropositivité n'a été observée selon le sexe (masculin versus féminin) pour le VIH (p = 0,344), le VHB (0,186) ou le VHC (0,734) au sein de notre population d'étude.

## Discussion

Les stratégies de triple élimination des infections virales majeures constituent des enjeux pour plusieurs pays africains, surtout dans des contextes d'endémies généralisées. Notamment, l'atteinte du contrôle de l'épidémie pour le VIH passe inévitablement par l'atteinte des trois 95 d'ici fin 2025 (*au moins 95% des personnes vivant avec le VIH connaissent leur statut VIH; au moins 95% des personnes qui connaissent leur statut VIH suivent un traitement ARV; au moins 95% des personnes sous traitement ont une charge virale supprimée*) selon les objectifs fixés par l'ONUSIDA [6], permettant ainsi d'éliminer toute nouvelle infection et la morbi-mortalité associées au VIH d'ici horizon 2030. Par ailleurs, l'élimination des hépatites virales B et C d'ici l'horizon 2030 concourt à la réduction des nouvelles infections, à l'augmentation du dépistage et du diagnostic, ainsi qu'au lien avec les services de prise en charge des personnes déclarées positives, avec un accent sur l'Afrique subsaharienne au regard de l'endémicité de ces infections et de la recrudescence des cas de carcinome hépatocellulaire telle que décrite par l'OMS dans sa stratégie de riposte mondiale [7].

La maîtrise des données épidémiologiques permet de définir les stratégies d'intervention selon la cible populationnelle et les ressources programmatiques nécessaires localement [8]. Ces pathologies virales transmissibles sont fréquentes au Cameroun et leur prévalence varie d'une zone géographique à une autre. La pertinence de cette étude réside dans sa couverture géographique dans une région ayant peu de données antérieures. De plus, la taille de l'échantillon est robuste (3188 participants) et renforce la fiabilité des résultats obtenus, et le lien systématique avec les services de prise en charge pour tous les cas positifs constitue une bonne pratique en faveur de l'atteinte des objectifs de l'élimination de ces pathologies. Concernant l'adhésion au testing de ces trois infections virales majeures durant notre étude, la quasi-totalité avait accepté le test et retiré les résultats par la suite. Ce résultat concorde avec celui de Ngangue *et al*. qui en 2016 à Douala au Cameroun avaient trouvé que les personnes qui subissent volontairement les tests de dépistage perçoivent un réel avantage et très peu d'inconvénients à connaitre leurs statuts [8].

Les séropositivités observées sont réparties de façon singulière au sein des groupes de population du Mayo-Tsanaga. La séropositivité au VIH était de 0,41%. Ce résultat tend à faire penser que la séropositivité au sein de cette population est inférieure aux moyennes nationales comme illustré au cours des enquêtes CAMPHIA 2017/2018 [9]. Les femmes représentent 77% des personnes testées positives. Ces résultats sont également en accord avec les rapports de l'OMS qui mettent en avant la tranche des 15-49 ans comme étant la plus touchée. La majorité des cas de VIH concernaient des femmes (77%), ce qui semble confirmer la tendance à la féminisation de l'épidémie observée dans d'autres contextes. Toutefois, cette différence n'était pas statistiquement significative dans notre échantillon, ce qui appelle à une interprétation prudente [10]. Ces résultats doivent appeler à l'action parce qu'il a été prouvé que même si la séropositivité ou la prévalence se stabilise ou baisse, le nombre de patients séropositifs continue d'augmenter.

Quant au VHB qui est endémique au Cameroun [11,12] nous avons obtenu une séropositivité de 11,7%. Cette valeur est bien plus élevée que les 8,3% retrouvés en 2021 dans une étude chez les donneurs de sang dans la région de l'Extrême-Nord ou encore les 6,1% de séropositivité nationale [13]. Ce résultat significativement élevé montre que la zone aurait sans doute une forte endémicité tout comme les pays voisins à l'instar du Nigeria [14]. Cette infection est surtout fréquente chez les plus de 50 ans. Ceci pourrait étayer la thèse d'une transmission très active au cours des campagnes contre certaines maladies tropicales dans les années 60 et aussi la transmission verticale qui s'en serait suivie [15]. Ce taux très élevé met également en exergue les observations faites par l'OMS qui avait tiré la sonnette d'alarme en rappelant que plus de 254 millions de personnes vivaient avec l'hépatite virale B et avait souligné la nécessité de mesures urgentes [16]. Bien que les femmes aient représenté une majorité des cas, aucune différence statistiquement significative n'a été mise en évidence selon le sexe.

Pour ce qui est de l'hépatite virale C, la séropositivité était de 0,31%. Ce taux reste inférieur aux estimations nationales qui sont comprises entre 2 et 10% selon les zones géographiques. Les cas étaient majoritairement observés entre 35 et 54 ans. Aucune association statistiquement significative n'a été observée selon le sexe ou l'âge. Au sein de notre population d'étude, nous constatons que le quart des infections est observé chez les personnes de plus de 50 ans, ce qui peut se superposer aux résultats de Kowo *et al*. en 2018 [15]. Ceci nous invite à renforcer les dépistages ciblés au sein des populations à risque.

Les tests d'association montrent qu'il n'y a pas de variation significative de séropositivité selon le sexe pour le VIH, le VHB ou le VHC dans cette population. Il n'y a pas d'association significative ici mais juste une tendance avec plus de femmes positives comme cela a été observé dans d'autres études menées au Cameroun [11,12]. Ces résultats semblent indiquer une circulation active des hépatites virales au sein de ces couches de population et sont d'autant plus inquiétants que selon l'OMS en 2021, seulement 2% des personnes infectées par le virus de l'hépatite B ont été diagnostiquées et à peine 0,1% d'entre elles ont été traitées. S'agissant de l'hépatite C, on estime que 5% des personnes infectées ont été diagnostiquées et que moins de 1% ont été traitées [17], dans un contexte où les coïnfections demeurent préoccupantes [18].

### Limites

Le nombre de patients positifs n'était pas très élevé et l'outil de collecte n'a pas inclus des données suffisantes pour mener des analyses plus poussées et ressortir notamment d'autres facteurs de risque décrits dans la littérature. Ainsi, ces aspects socio-comportementaux contribuant à l'endémicité du VHB devront être mieux abordés dans les futures investigations.

## Conclusion

A l'Extrême-Nord du Cameroun, la faible circulation du VIH et du VHC suggère une dynamique encourageante vers leur élimination. En revanche, le VHB demeure fortement endémique, vraisemblablement chez les personnes âgées. Ces évidences soulignent l'importance de renforcer les campagnes de vaccination, les efforts de sensibilisation et de dépistage ciblant prioritairement les jeunes, sous réserve des données complémentaires pour des actions de santé publique davantage probantes au niveau populationnel.

### Etat des connaissances sur le sujet

- Le taux de séropositivité au VIH et au virus de l'hépatite C était faible dans la région septentrionale du Cameroun, avec peu de données en stratégie communautaire;
- L'hépatite B était endémique au Cameroun et nécessitait une maîtrise de la situation épidémiologique en septentrion pour définir les interventions;
- Les données demeurent insuffisantes sur la co-circulation des trois infections (VIH, VHB, VHC) en communauté au Cameroun.

### Contribution de notre étude à la connaissance

- La séropositivité du VIH est faible dans le septentrion, confirmant ainsi l'efficacité des programmes de prévention mis en place;
- La très faible séropositivité du virus de l'Hépatite C suggère une évolution vers l'élimination dans la région septentrionale du Cameroun;
- La séropositivité du VHB est élevée et nécessite une priorisation des interventions en matière de prévention (vaccination de masse) et de traitement.

## References

[1] Organisation mondiale de la Santé VIH et sida. Organisation mondiale de la Santé Génève, 2022.

[2] UNAIDS Fiche d'information - Dernières statistiques sur l'état de l'épidémie de sida ONUSIDA (2024). Génève.

[3] Keugoung B, Ymele FF, Mabou JD, Nangue C, Kougoum PN, Takoudjou L (2013). Financement d'une campagne de soins gratuits par une association dans un district rural au Cameroun Nécessité d'optimiser le rôle de la société civile en Afrique subsaharienne. Med Sante Trop.

[4] Ondigui JLN, Kenmoe S, Kengne-Ndé C, Ebogo-Belobo JT, Takuissu GR, Kenfack-Momo R (2022). Epidemiology of occult hepatitis B and C in Africa: A systematic review and meta-analysis. J Infect Public Health.

[5] Taverne B, Desclaux A, Delaporte E, Ndoye I, Coll Seck AM, Barré-Sinoussi F (2013). Universal health coverage and HIV in resource-constrained countries: a critical juncture for research and action. AIDS.

[6] UNAIDS (2024). Comprendre les mesures des progrès réalisés pour atteindre les objectifs 95-95,95 en matière de dépistage, de traitement et de suppression virale du VIH. ONUSIDA, Génève.

[7] OMS Journée mondiale contre l'hépatite 2022: des soins anti-hépatite de plus grande proximité. Organisation mondiale de la Santé, Génève, 2022.

[8] Ngangue PA, Gagnon MP, Bedard E (2016). Return for HIV test results after voluntary screening in Cameroon. Santé Publique.

[9] Ministère de la Santé Division de la Recherche Opérationnelle en Santé (DROS), Comité National de Lutte contre le Sida (CNLS) Institut National de la Statistique (INS) Le Plan d'urgence du Président des États-Unis pour la lutte contre le SIDA (PEPFAR) Les centres des États-Unis pour le contrôle et la prévention des maladies (CDC) ICAP de l'Université de Columbia (2020). Evaluation de l’impact du VIH sur la Population au Cameroun CAMPHIA 2017-2018. Rapport final décembre.

[10] ONUSIDA (2022). Rapport mondial actualisé sur le sida. ONUSIDA, Génève.

[11] Bigna JJ, Amougou MA, Asangbeh SL, Kenne AM, Noumegni SRN, Ngo-Malabo ET (2017). Seroprevalence of hepatitis B virus infection in Cameroon: a systematic review and meta-analysis. BMJ Open.

[12] Hamadou NHM, Njoya O, Kowo MP, Ankouane F, Talla P, Babagna ID (2018). Traitement de l'Hépatite C de Génotype 1 par les Antiviraux d'Action Directe au Cameroun: Résultats Préliminaires. Health Sci Dis.

[13] Comité National de Lutte contre le Sida Plan Stratégie National de lutte contre le VIH. Sida et les IST, Comité National de Lutte contre le Sida, septembre 2023.

[14] Essi MJ, Mamouda MN, Nguizaye L, Ondoua R, Hopp E, Penda R (2018). Compétences de Santé Vis-à-vis de l'Hépatite Virale B dans les Camps de Réfugiés au Cameroun. Health Sci Dis.

[15] Kowo MP, Ngankhoué OM, Ankouane F, Ndam AN, Talla P, Ouamba PG (2018). Profil Épidémiologique des Personnes Récemment Infectées par le Virus de l'Hépatite C au Cameroun. Health Sci Dis.

[16] World Health Organization Global hepatitis report 2017. World Health Organization.

[17] OMS (2024). L'OMS en Afrique: 91 millions d'Africains infectés par l'hépatite B ou C. OMS. Bureau régional pour l'Afrique. OMS.

[18] Bigna JJ, Nkeck JR, Ngouo A, Nyaga UF, Noubiap JJ (2018). Hepatitis B virus and HIV coinfection among adults residing in Cameroon: A systematic review and meta-analysis of prevalence studies. Infect Dis Health.

